# Patterns and perceptions of supplement use among patients with plasma cell disorders

**DOI:** 10.1038/s41408-026-01603-x

**Published:** 2026-08-14

**Authors:** Maria A. Malik, Eliana Schach, Yan Leyfman, Andriy Derkach, Francesca Castro, Jorge Arturo Hurtado Martinez, Ana M. Sahagun Sanchez Aldana, Patricia Alejandra Flores Perez, Jennifer M. Ahlstrom, Jay R. Hydren, Saad Z. Usmani, Jun J. Mao, Susan Chimonas, Urvi A. Shah

**Affiliations:** 1 Department of Medicine, NYU Grossman School of Medicine, New York, NY USA; 2 Montefiore Medical Center, Albert Einstein College of Medicine, Bronx, NY USA; 3 Department of Hematology and Oncology, Meyer Cancer Center, New York Presbyterian Brooklyn Methodist Hospital, Brooklyn, NY USA; 4 Department of Epidemiology and Biostatistics, Memorial Sloan Kettering Cancer Center, New York, NY USA; 5 Myeloma Service, Department of Medicine, Memorial Sloan Kettering Cancer Center, New York, NY USA; 6 HealthTree Foundation, Lehi, UT USA; 7 Integrative Medicine Service, Memorial Sloan Kettering Cancer Center, New York, NY USA; 8 Center for Health Policy and Outcomes, Memorial Sloan Kettering Cancer Center, New York, NY USA

**Keywords:** Myeloma, Myeloma

## Introduction

As novel treatments have improved survival in patients with plasma cell disorders (PCDs), there is a growing interest in complementary medicine (CM), particularly dietary supplements (vitamins, minerals, antioxidants, and herbs), amongst patients with cancer [1]. Multiple myeloma (MM) and its precursor conditions (monoclonal gammopathy of undetermined significance (MGUS) and smoldering multiple myeloma (SMM)) provide opportunities for CM, as the standard practice for precursor conditions is observation while MM requires long term treatment – during which supportive strategies may improve treatment tolerance, response, and quality of life [2].

Current recommendations from the National Comprehensive Cancer Network (NCCN) and American Cancer Society (ACS) do not support routine supplement use in cancer, except for certain vitamin deficiencies or specific clinical indications [3]. However, several large-scale surveys and cohort studies have found that supplement use is common (about 70%) amongst cancer survivors, the most frequent being multivitamins, vitamin D, and omega-3 [4, 5]. Despite widespread use, 59% of oncologists report having limited education on supplements, which is a barrier to effectively counsel patients and a missed opportunity to strengthen patient-provider trust [6]. More concerningly, inadequate supplement knowledge among oncologists may compromise patient outcomes. For example, certain antioxidant supplements worsen chemotherapy effectiveness and are associated with lower survival in patients with breast cancer, and preclinical studies suggest vitamin C and green tea extract can reduce the effectiveness of the MM drug Bortezomib [7–9].

While patients with PCDs have reported use of nutrition and natural products, there remains a substantial gap in research on dietary supplement use in this population [10, 11]. The aim of this survey was to examine supplement use patterns, perceptions, interest in medical guidance and research, and to identify areas for future investigation in participants with PCDs. The study focused on four supplements commonly used by patients with PCDs – vitamin D, curcumin, omega-3 and probiotics.

## Subjects and methods

A 23-question online survey exploring supplement use (including pre- and post-diagnosis usage patterns) and perceptions was distributed to approximately 9000 registered users with a self-reported PCD diagnosis via the HealthTree® Foundation’s Patient Portal platform (with email reminders) from September 2023 to May 2024, although the number of active users on the site is unknown [10, 12]. This study was approved by the Memorial Sloan Kettering Cancer Center (MSK) Institutional Review Board and determined to be exempt from further review. Participants provided electronic informed consent at survey initiation. Deidentified survey responses and pre-collected demographic and health data were retrieved from the HealthTree platform at study conclusion. Summary statistics were used to assess the distribution of responses based on the number of complete responses for each given question. Fisher’s exact test and Chi-square test of independence were applied for pre- and post-diagnosis comparisons.

## Results

The survey had 563 respondents (approximately 6.3% response rate): 60% female, 59% ≥ 65 years, 79% White, 68% with at least a college degree, and 73% with MM (Table 1). The proportion of participants reporting taking at least one supplement increased after a PCD diagnosis compared with before diagnosis (96% vs 72%; *p* < 0.00001). Participants also reported taking a greater number of supplements post PCD diagnosis – for instance, the proportion taking ≥6 supplements increased from 12% to 34% (*p* < 0.00001). Younger patients (≤ 50 years) were more likely to increase supplement use compared to older patients (*p* < 0.005). There was no difference for sex, race, education, or type of PCD diagnosis (MM vs precursor disorder) in supplement use pre- versus post-diagnosis.

**Table 1:** Characteristics of study participants and changes in supplement use post diagnosis.

Characteristics	Study Participants *n* = 563 (%)	Increase in Supplement Use Post Diagnosis^a^ *n* = 258	Decrease/No Change in Supplement Use Post Diagnosis^a^ *n* = 305	*p*-value
**Sex**				
Female	340 (60)	160 (47%)	180 (53%)	
Male	223 (40)	98 (44%)	125 (56%)	0.490
**Age**				
≥ 65 years	330 (59)	137 (42%)	193 (58%)	
51 – 64 years	196 (35)	96 (49%)	100 (51%)	**<0.005**
≤ 50	37 (7)	25 (68%)	12 (32%)	
**Race**^**b**^				
White	447 (79)	207 (46%)	240 (54%)	0.677
Non-White	34 (6)	51 (44%)	65 (56%)	
Black or African American	25 (4)	-	-	
Asian	6 (1)			
American Indian/Alaska Native	3 (1)			
Unknown or Did Not Answer	82 (14)			
**Education**				
College Degree and Above	382 (68)	177 (46%)	208 (54%)	
Some College	70 (12)	32 (46%)	38 (54%)	
Vocational or Technical School	18 (3)	9 (50%)	9 (50%)	
High School Graduate	11 (2)	5 (45%)	6 (50%)	
Some High School	2 (0)	1 (50%)	1 (50%)	0.993
Unknown or Did Not Answer	80 (14)	-	-	
**Plasma Cell Disorder Diagnosis**				
Multiple Myeloma	413 (73)	191 (46%)	222 (54%)	
Precursor Disorder (SMM/MGUS)	56 (10)	25 (45%)	31 (55%)	0.887
Other or Unknown^c^	94 (17)	42 (45%)	52 (55%)	
		**Pre-Diagnosis**	**Post-Diagnosis**	***p*-value**
**Taking at Least One Supplement (*n*** = **563)**		405 (72%)	538 (96%)	**<0.00001**
**Number of Supplements Taken (*n*** = **563)**				
0		158 (28%)	25 (4%)	**<0.0001**
1-5		338 (60%)	349 (62%)	0.502
≥6		67 (12%)	189 (34%)	< **0.00001**

^a^Percentage calculated based on the total number of respondents in each *Characteristics* sub-category

^b^Note: this study included numerous international participants – Unites States (*n* = 510), Canada (*n* = 13), Australia (*n* = 7), United Kingdom (*n* = 6), Netherlands (*n* = 3), France (*n* = 2), Italy (*n* = 2), Sweden (*n* = 2), Bosnia and Herzegovina (*n* = 1), Brazil (*n* = 1), Germany (*n* = 1), Hong Kong (*n* = 1), India (*n* = 1), Israel (*n* = 1), Mexico (*n* = 1), New Zealand (*n* = 1), Portugal (*n* = 1), South Africa (*n* = 1), Spain (*n* = 1), and Unknown (*n* = 6).

^c^Among these 94 patients, 4 had AL amyloidosis, 1 had Waldenström macroglobulinemia, 25 had plasma cell disorders with unlinked diagnosis dates that limited data extraction, and the remaining 63 had no diagnosis information recorded in HealthTree and may represent individuals registered through partner channels or non-patient respondents.

The number of participants taking vitamin D (87% vs 47%), curcumin/turmeric (47% vs 19%) or probiotics (47% vs 36%) was significantly higher post-diagnosis than pre-diagnosis (*p* < 0.001) and there was no difference in omega3 use (33% vs 34%) (Supplementary Table 1). Though most (67%) reported that their doctors checked their vitamin D levels, 65% had indicated they were unsure what the level was or did not remember being tested; 10% reported low levels and 26% reported normal or high levels. Compared to patients with MM, patients with MGUS or SMM were more likely to take omega-3 (46% vs 32%; *p* < 0.05) and curcumin/turmeric (66% vs 42%; *p* < 0.001), and there was no significant difference in vitamin D (89% vs 88%) and probiotics (55% vs 45%) between these two groups (Supplementary Table 1). The majority of patients perceived no change in symptoms after taking these supplements while a smaller percentage noticed improvements or worsening of their symptoms (Supplementary Table 2).

Common motivations for taking supplements after PCD diagnosis included for immune support (70%), to prevent nutritional deficiencies (53%), to mitigate disease progression (39%), and to better tolerate chemotherapy (17%) (Table 2). Most respondents (77%) did not feel that supplements added to their daily pill burden. Most (61%) reported that their spending on supplements increased post-diagnosis, with 63% spending greater than $20 per month and 14% reporting increased financial burden due to supplement costs.

**Table 2:** Overall supplement use trends and attitudes in patients with plasma cell disorders.

Survey Question or Topic	*n*	%
**Overall Perceptions of Supplement Use and Cost**		
**What motivated you to take supplements after your plasma cell disorder diagnosis? Select all that apply** ***n*** = **563**		
To support my immune system	394	70
To prevent nutritional deficiencies	300	53
To stop cancer progression or prevent cancer	221	39
To tolerate chemotherapy better	97	17
**Do you feel that taking supplements adds to your daily pill burden?** ***n*** = **563**		
Yes	99	17
No	439	77
Not applicable	35	6
**Approximately how much do you spend per month on supplements?** ***n*** = **552**		
$0		
$1–20	10	2
$21–50	192	35
$51–100	183	33
>$100	81	15
**Since being diagnosed, has your spending on supplements:** ***n*** = **547**		
Increased	332	61
Stayed the same	188	34
Decreased	27	2
**Has the increased spending on supplements caused a financial burden on you or on your family?** ***n*** = **332**		
No	286	86
Yes	46	14
**Information Sources and Interest in Further Research Regarding Supplements**		
**Where do you get information about supplements? Select all that apply.** ***n*** = **563**		
Hematologist/Oncologist	261	46
Online Medical Resources	260	46
Primary Care Provider	201	36
Myeloma Education Websites	159	28
Online Patient Support Forums	115	20
Dietician/Nutritionist	92	16
**After your diagnosis, did your doctor answer your questions about supplements, if you had any?** ***n*** = **559**		
Yes	357	64
No	202	36
**Did you follow the advice of a physician or nutritionist/dietitian regarding supplement use?** ***n*** = **563**		
Yes	327	58
No	34	6
N/A	202	36
**What advice did your hematologist/oncologist give you about supplement use? Select all that apply.** ***n*** = **563**		
Recommended I take a specific supplement	240	43
None - my hematologist/oncologist did not discuss supplement use with me	155	28
Told me to stop taking specific supplements	87	15
Told me about the risks and benefits of supplement use	84	15
Referred me to a nutritionist/dietitian/naturopath/integrative medicine specialist	70	12
**Would you like your hematologist/oncologist to give you recommendations about supplement use?** ***n*** = **557**		
Yes	416	75
No	53	10
Not sure	88	16
**Are you interested in learning about research on any risks and benefits of supplements for patients with plasma cell disorders?** ***n*** = **563**		
Yes	512	91
No	22	4
Not sure	29	5

Common sources to receive information about supplements included a hematologist/oncologist (46%), online medical resources (46%), primary care provider (36%), myeloma education websites (28%), and online patient support forums (20%). Over half (58%) of patients reported that they followed the advice of a physician, dietician, or nutritionist regarding supplement use. Advice from hematologists/oncologists included recommendations to take a specific supplement (43%), instructions to stop taking specific supplements (15%), and risk/benefit discussions regarding supplement use (15%).

Overall, most respondents (75%) indicated that they would want to receive recommendations regarding supplement use from their hematologist/oncologist. The vast majority (91%) expressed interest in learning about research on the risks and benefits of supplement use and their plasma cell disorder diagnosis.

## Discussion

To our knowledge, this is the largest observational study on supplement use patterns in PCDs. Supplement use was nearly universal (96%) with a significant increase in intake post-diagnosis. This is consistent with prior research showing supplement use is more prevalent among cancer survivors compared to individuals without cancer [4]. However, the prevalence observed in our study appears higher than previously reported, possibly due to widespread utilization of vitamin D; prolonged disease and treatment courses that may increase interest in self-directed health strategies; and self-selection bias inherent to survey-based research [13].

Younger age emerged as the only demographic factor associated with increased supplement use, which may reflect different treatment experiences (i.e., receiving more aggressive therapies) and unique psychosocial patterns (i.e., more likely to utilize online health information and be more proactive in self-directed health management) [14]. Interestingly, there was no difference post-diagnosis between patients with cancer (MM) versus a precursor disorder, suggesting similar motivation to integrate CM strategies. Most patients did not perceive supplements to contribute substantially to pill burden or cause financial strain, indicating that patients view supplements as accessible and integrate it into routine care.

Most of the commonly taken supplements (vitamin D, curcumin/turmeric, and probiotics) saw increases in usage following diagnosis. We also noted that patients with MGUS/SMM were taking omega-3 and curcumin/turmeric supplements at significantly higher rates than patients with MM, suggesting this population may be especially motivated to integrate CM strategies to modify disease progression. Similarly, long-term follow-up data from a randomized controlled trial of curcumin use and MGUS/SMM progression suggests sustained curcumin use, well beyond study completion [15, 16].

Although 87% of patients reported taking vitamin D after diagnosis, only 67% indicated that their serum levels had been checked by a clinician, signifying that many patients were supplementing without an established baseline. Vitamin D deficiency is common among patients with MM, and current NCCN guidelines recommend assessing vitamin D status primarily in patients receiving bone-targeting or steroid-containing therapies which nearly all patients treated for MM receive. Further, vitamin D deficiency is associated with worse survival in MM although randomized prospective trials have not been done [13, 17]. Hematologists and oncologists should consider checking vitamin D levels at baseline and periodically in this population to better guide dosage.

This study highlights a clear opportunity for hematologists and oncologists to play a more active role in guiding dietary supplement use, as 75% of patients expressed interest in receiving recommendations regarding supplement use from their provider and 91% wished to learn more about research on the risks and benefits of supplement use in the context of their disease. Prior research has documented substantial communication gaps between patients with cancer and clinicians regarding CM use, with patient non-disclosure rates as high as 77% [1, 18]. Barriers include concerns about negative provider reactions, perceived limited provider knowledge, and lack of clinician-initiated discussion. Given that inappropriate supplement use may adversely affect outcomes and that medical providers were the most frequently cited sources of supplement-related information in our survey, it is imperative for hematologists/oncologists to proactively engage in informed discussions about supplements with their patients. The MSK website/app “About Herbs” and the National Center for Complementary and Integrative Health’s database “Herbs at a Glance” are reliable resources that can be shared with healthcare professionals and patients [19, 20].

Strengths of this study include utilization of the HealthTree Portal which enabled wide international outreach and robust participation (Table 1). Limitations include an overrepresentation of White participants despite MM disproportionately affecting Black individuals. Additionally, the study relied on self-reported data which may be affected by recall bias, overestimating effect size when comparing supplement pre-diagnosis to post. Results may be affected by selection bias from patients interested in this topic and in using supplements and complementary medicine as well as from those more technologically adept given it was administered online. The survey focused on four specific supplements and did not systematically assess all supplements used by participants. We were therefore unable to characterize which other supplements patients were taking and it is unclear whether commonly recommended supplements, particularly multivitamins, may be driving the high prevalence of supplement use. Finally, the survey did not evaluate patients’ knowledge of potential drug interactions between supplements and cancer therapies, an important area for future investigation.

## Conclusions

Supplement usage is nearly universal among patients with PCDs, with the majority increasing usage post-diagnosis. Despite widespread use, there is a lack of clear indication along with inadequate monitoring, highlighting gaps in clinical guidance. There are no established guidelines or current clinical studies that directly address the benefits of supplements for plasma cell disorders, though ongoing NUTRIVENTION trials (NCT05640843 and NCT06055894) are exploring certain supplements in precursor PCDs. Other complementary approaches, such as a high-fiber, plant-based dietary intervention (NUTRIVENTION), are being studied in this population [21]. Our findings emphasize the need for further supplement research in PCDs.

## Supplementary information

Supplemental Table 1

Supplemental Table 2.

Reproducibility checklist

## Data Availability

The datasets generated during and/or analyzed during the current study are available from the corresponding author on reasonable request.
