# Supplementary material for: Patterns and perceptions of supplement use among patients with plasma cell disorders
Source: Blood Cancer J. 2026 Aug 14;16(1):137. doi: 10.1038/s41408-026-01603-x (PMC13473143; doi:10.1038/s41408-026-01603-x)
Supplement: Supplementary file 2 [file 41408_2026_1603_MOESM1_ESM.docx]

**Supplementary Table 1: Supplement Specific Intake Patterns in Patients with Plasma Cell Disorders**

| **Supplement Specific Intake Patterns** | | | | | | | | | |
| --- | --- | --- | --- | --- | --- | --- | --- | --- | --- |
| **Supplement Type** | **Taking Pre- Diagnosis**  **(n = 563)** | **Taking Post-Diagnosis**  **(n = 563)** | **p-value** | **Increased Dose or Frequency Post- Diagnosis*** | **No Change Post- Diagnosis*** | **Eventual Dose Decrease/Stop Post Diagnosis*** | **MM**  **(n = 413)** | **MGUS/**  **SMM**  **(n = 56)** | **p-value **** |
|  |  |  |  |  |  |  |  |  |  |
| **Vitamin D** | 263 (47%) | 489 (87%) | **<0.00001** | 234 (48%) | 229 (47%) | 26 (5%) | 363 (88%) | 50 (89%) | 0.763 |
| **Omega-3** | 193 (34%) | 186 (33%) | 0.7052 | 64 (34%) | 104 (56%) | 20 (11%) | 131 (32%) | 26 (46%) | **0.029** |
| **Curcumin/Turmeric** | 106 (19%) | 262 (47%) | **<0.00001** | 138 (53%) | 78 (30%) | 46 (18%) | 175 (42%) | 37 (66%) | **0.0008** |
| **Probiotics** | 203 (36%) | 265 (47%) | **0.0002** | 105 (40%) | 122 (46%) | 38 (14%) | 187 (45%) | 31 (55%) | 0.156 |

* Percentages calculated based on total respondents that had had indicated they were taking the supplement post-diagnosis in a preceding question: Vitamin D n= 489; Omega-3 n= 186; Curcumin/Turmeric n= 262; Probiotics n= 265.

** Comparison of patients with MM vs MGUS/SMM who reported taking the supplement “after” or “before and after” their diagnosis; patients with other/unknown diagnoses (n=94) were excluded from this analysis.
