# Supplementary material for: Patterns and perceptions of supplement use among patients with plasma cell disorders
Source: Blood Cancer J. 2026 Aug 14;16(1):137. doi: 10.1038/s41408-026-01603-x (PMC13473143; doi:10.1038/s41408-026-01603-x)
Supplement: Supplementary file 3 [file 41408_2026_1603_MOESM2_ESM.docx]

**Supplementary Table 2: Perceived Supplement Specific Benefits in Patients with Plasma Cell Disorders**

| **Perceived Supplement Specific Benefits After Diagnosis** | | | | |
| --- | --- | --- | --- | --- |
|  | **Symptom Improved** | **No Change in Symptom** | **Symptom Worsened** | **Symptom Not Experienced** |
| **Vitamin D (n = 489)** | | | | |
| Low 25-Hydroxy Vitamin D Levels | 83 (17%) | 97 (20%) | 6 (1%) | 283 (58%) |
| Depression or Mood Changes | 23 (5%) | 202 (42%) | 20 (4%) | 231 (47%) |
| Joint or Bone Pain | 43 (9%) | 215 (44%) | 42 (9%) | 307 (36%) |
| Low Energy Level | 39 (8%) | 246 (50%) | 46 (9%) | 146 (30%) |
| Frequent Respiratory Infections | 42 (9%) | 162 (33%) | 37 (8%) | 235 (48%) |
| **Omega-3 (n=186)** | | | | |
| Depression or Mood Changes | 5 (3%) | 92 (49%) | 6 (3%) | 82 (44%) |
| Joint or Bone Pain | 17 (9%) | 91 (49%) | 10 (5%) | 64 (35%) |
| Memory Changes | 13 (7%) | 89 (48%) | 19 (10%) | 63 (34%) |
| Vision Changes | 9 (5%) | 95 (51%) | 23 (12%) | 55 (29%) |
| Triglyceride Levels | 23 (12%) | 94 (51%) | 4 (2%) | 63 (34%) |
| Average Blood Pressure Measurements | 14 (8%) | 100 (54%) | 11 (6%) | 58 (31%) |
| **Curcumin/Turmeric (n=262)** | | | | |
| Depression or Mood Changes | 17 (6%) | 125 (47%) | 5 (2%) | 113 (43%) |
| Joint or Bone Pain | 51 (19%) | 99 (37%) | 19 (7%) | 89 (33%) |
| Memory Changes | 15 (6%) | 133 (51%) | 27 (10%) | 81 (31%) |
| Average Blood Pressure Measurements | 17 (6%) | 141 (53%) | 17 (6%) | 80 (31%) |
| Digestive Health | 25 (10%) | 128 (49%) | 25 (10%) | 73 (27%) |
| **Probiotics (n=265)** | | | | |
| Bloating or Flatus Changes | 76 (29%) | 105 (39%) | 27 (10%) | 54 (20%) |
| Diarrhea | 62 (23%) | 95 (36%) | 23 (9%) | 80 (20%) |
| Urinary Tract Infections | 12 (5%) | 90 (34%) | 6 (2%) | 148 (56%) |
| Mental Fog | 17 (6%) | 117 (44%) | 16 (6%) | 106 (40%) |
